# Charlotte Pommer: Resistance fighter and female pioneer of German anatomy

**DOI:** 10.1002/ase.70268

**Published:** 2026-05-28

**Authors:** Tim S. Goldmann

**Affiliations:** ^1^ Institute for History and Ethics of Medicine FAU Erlangen‐Nürnberg Erlangen Germany

**Keywords:** Charlotte Pommer, gender and science, history of anatomy, women in science and medicine

## Abstract

This article examines the biography and unique case of Charlotte Pommer (1914–2004), the only anatomist documented to have left the field during the Nazi period after encountering the regime's victims on the dissection table. While she is known for her resistance activities, newly presented documentation reveals her role as the provisional director of the Institute of Anatomy in Erlangen from 1947 to 1949. Consequently, she is likely the first woman in Germany to hold such a leadership position, albeit not as a full professor. Based on an analysis of archival sources and her personal correspondence, this study reconstructs Pommer's biography, institutional role, and life after 1945. It demonstrates that her early engagement with the resistance against the Nazi regime and the responsibilities she assumed as a young physician fundamentally shaped her worldview. These findings contribute to the historiography of women in academic science and anatomy and provide a significant case study of a contemporary witness to the Nazi era. Her late reflections illustrate that the psychological burdens and ethical dilemmas of her early career remained unresolved in old age. By interpreting her life as part of an “ongoing war among Germans,” Pommer transformed personal experience into a historical‐moral commentary on post‐war German identity. This dual perspective—documented leadership combined with a self‐authored testimony of moral struggle—renders Pommer not only a pioneering figure in German anatomy but also a witness whose medical and moral self‐conception bridges the ruptures of the mid‐twentieth century and turbulent post‐war eras in Germany.

## THE CASE OF CHARLOTTE POMMER AND ANATOMY IN THE 20TH CENTURY

In recent years, historical research has increasingly turned toward the role of women in science and medicine. Much of this work has emphasized the structural barriers they faced, the limited opportunities for professional recognition, and the strategies women developed to navigate exclusionary institutions, specifically within the field of anatomy.^1^, ^2^ While the histories of prominent female anatomists, scientists, and physicians have received some attention, far less has been written about those who worked at the margins of established societies and disciplines—often without formal membership or institutional support—or those who worked in specific fields for only a brief duration.

Among these figures is Charlotte Pommer, whose biography merits public recognition. She initially gained renown for her role in the resistance during the Nazi era.^3^, ^4^ ^(p11)^ Furthermore, recent discoveries indicate that in 1947 Pommer exercised de facto management of the Erlangen Institute of Anatomy. She therefore stands among the earliest clearly documented women to exercise operational leadership in a German anatomy institute, alongside, for example, Auguste Hoffmann (1902–1989), who was official interim director at the anatomical institute in Greifswald 1 year later in 1948. However, a systematic national survey would be required to establish whether Pommer was the first. This finding prompted further research into her post‐1945 biography, yielding new results regarding the history of women in anatomy, the institutional history of Friedrich‐Alexander‐Universität Erlangen‐Nürnberg (FAU), and Charlotte Pommer herself.

Building upon the initial description of her position in Erlangen, this article presents new insights into her post‐1945 career, her family, her correspondence with prominent historian Golo Mann (1909–1994, son of German author and Nobel laureate Thomas Mann), and her relationship with her doctoral supervisor, pharmacologist Wolfgang Heubner (1877–1957). These findings aim to illuminate the intellectual landscape of a female physician who participated in the resistance against the Nazi regime and subsequently navigated the complexities of a transforming post‐war German society. These findings also facilitate an understanding of Charlotte Pommer's worldview, encouraging a critical reflection on her own accounts—first published in Barbara Orth's Book *Gestapo im OP* in 2013—through archival and external sources. The new results presented here include a tenure in Canada at the General Hospital Oshawa in Ontario; her activities in the USA, Landstuhl, Switzerland, and England; and information about her family.

### Sources and methodology: Tracing the life of an elusive woman

Charlotte Pommer's life and work have been documented and acknowledged in varying detail since the first scholarly mention by Winkelmann and Schagen,^5^ wherein her autobiographical manuscript—later edited by Orth—was cited as “Pommer, 1967.” Subsequent references include Vollmer,^6^ ^(p292f.)^; Orth^3^; Tuchel^4^; Hildebrandt^7^; Glauer and Knapp^8^ and Winkelmann.^4^ The earliest references to Pommer date back to Bidermann^9^ ^(p285f.)^; Zur Mühlen and Bauer^10^ ^(p76f.)^; Alvensleben^11^ ^(p109f.)^ and Reynolds.^12^ ^(p85)^ However, she was mentioned only briefly in these works. Barbara Orth (and Charlotte Pommer herself) detailed the stages of her life in *Gestapo im OP*, and most recently, she was discussed by Lohmann.^13^ While these works focus on her life up to 1945, this article examines her later life, her family, and her worldview in greater detail.

Data regarding Charlotte Pommer were primarily derived from files in the *Universitätsarchiv Erlangen* (*UAE*, University Archive Erlangen) and *Berlin* (*UAH*, Humboldt University Archive), the *Bayerisches Hauptstaatsarchiv* (*BHStA*, Bavarian Main State Archive) in Munich and the *Bundesarchiv* (*BArch*, Federal Archives) in Berlin, the *Landesarchiv Berlin* and *Staatsarchiv Hamburg* (*LAB*, Berlin State Archive and *StAH*, Hamburg State Archive), the National Archives of the United States in Washington DC (*NA*), the Max Planck Society Archive (*AMPG*), the Swiss Literary Archive (*SLA*)—where her correspondence with Golo Mann is preserved—the German National Library (*DNB*), and the Arolsen Archives (*AA*). Additionally, sources include her autobiographical account published in Orth,^3^ kept at the *Institut für Zeitgeschichte München* (Institute for Contemporary History in Munich), and the diaries of Wolfgang Heubner, transcribed by Erich Muscholl (Mainz), housed in the *Archive of the Deutsche Gesellschaft für Experimentelle und Klinische Pharmakologie und Toxikologie* (*ADGPT*, German Society for Experimental and Clinical Pharmacology and Toxicology). Personal correspondence of the author with other researchers, state archives and local authorities further aided in exploring her biography. All translations from German sources into English were carried out by the author.

Despite attempts to locate witnesses and contemporaries, such as colleagues or friends, of Charlotte Pommer in Germany and the US, no living persons who knew her personally could be found. While relatives of individuals who knew Pommer—or her close associates—were located, these inquiries often yielded no further information; in instances where relevant data was obtained, it is noted accordingly within the text. Also, despite extensive searches in international archives and estates, no verifiable photographs of Charlotte Pommer could be located as of now. Thus, one aim of this manuscript is to draw the attention of such persons to Pommer's importance and to share their story in the future, in the hope of possibly finding further information and a photograph of her.

## CHARLOTTE POMMER'S LIFE AND CAREER

### Early life, family and medical education (1914–1941)

Martha Brigitte Charlotte Pommer was born on November 9, 1914 in Berlin, the daughter of the bookseller and authorized signatory Karl Heinrich Walter Pommer (November 30, 1880, Leopoldshall–January 21, 1946, Berlin) and his wife Helene Marie Frieda, née Eichhorn (March 17, 1880, Berlin–July 12, 1945, Berlin). Her parents, Walter and Frieda, married on August 24, 1907 in Berlin‐Schöneberg. Little is known of them beyond the fact that in 1939 Walter Pommer became a shareholder of *Wilhelm Ernst & Sohn, Gropius'sche Buch‐ und Kunsthandlung*, and *Buchdruckerei Gebrüder Ernst* (DNB, Bö‐GR/E/489).

Charlotte Pommer had an older brother, Ulrich Adolf Helmut Pommer (December 3, 1908, Berlin–April 8, 1986, Palma/Spain). Their parents reported him missing in 1927, as he disappeared from home on June 30, 1927 and never came back.^14^ At the time, he had told his classmates that he wanted to go abroad, particularly to Austria. He was in his final year of secondary school, so he was about to take his A‐levels. In fact, he soon served as a soldier in the Wehrmacht, was stationed in southern Germany and Austria, where he apparently went after he disappeared, and was taken prisoner by the Americans toward the end of the war (BArch, B 563/56796). In 1955, Helmut emigrated to the United States with his wife Ingeborg (1925–2017) and their two children. Although the family continues to reside in the United States, they had no knowledge of Charlotte Pommer's later life, suggesting that the siblings never had contact again after he disappeared.^a^ The reason why remains unknown. A parallel between them is that both lived in the US for a time and converted to Catholicism, though this appears to be coincidental.^15^

Until 1928, Charlotte Pommer attended the *Auguste‐Viktoria School* in Berlin‐Steglitz, followed by the *Königin‐Luise School* in Berlin‐Friedenau until Easter 1934.^16^ ^(p83)^ No archival records relating to Charlotte Pommer could be located in the school archives of the *Paul Natorp Gymnasium* (the successor to the Königin‐Luise School) or the *Auguste‐Viktoria School* except her *Reifezeugnis* (Certificate of general university entrance qualification) (LAB, A Rep. 020‐46, Nr. 23). The only other mention of her during this time is the reward of a special book prize for her “humorous verses and musical elaborations” from a journal in 1933 (*Neue Zeitschrift für Musik*, New Journal of Music, Bosse^17^ ^(p252)^) This award demonstrates her early connection to music, a theme she reflects upon in her manuscript published in Orth.^3^

During her medical studies, which she began in 1936, Charlotte Pommer obtained her doctorate at the Pharmacological Institute under Wolfgang Heubner with a thesis entitled “On the Effect of Certain Azulenes on Inflammation” on November 17, 1941, which she also published in 1942 in the “Naunyn‐Schmiedeberg Archive for Experimental Pathology and Pharmacology”.^16^, ^18^ She completed her medical studies in Berlin shortly before this, on 19 March 1941. Throughout this period, she resided at her parents' address at Florastraße 15 in Berlin‐Steglitz until 1941, when she moved to Südendstraße 15 as subtenant (LAB, C Rep. 031‐02‐12 Nr. 81).

### The Berlin period: Conscientious objection and resistance (1941–1945)

After her medical studies, she undertook brief tenures as a *Pflichtassistentin* (mandatory assistant, a necessary period of work to recognize the successful completion of her medical studies) at the Bethanien Hospital in Berlin and the Ebenhausen Sanatorium near Munich (see Table 1) (StAH, 221‐11, M. 2601). In September 1941, she commenced work at the Institute of Anatomy in Berlin under the direction of Hermann Stieve, where she, among other duties, directed the “Ladies' Hall of the Muscle Department,” effectively co‐supervising the female students in the dissection course (HU UA, Bestand PA nach 1945, Stieve, Hermann). She remained in this position until 31 March 1943, at which point she resigned voluntarily.

**TABLE 1:** Biographical timeline of Charlotte Pommer (1914–2004).

Period	Location	Institution/context	Position/activity	Biographical & historical significance
1914–1934	Berlin, Germany	Auguste‐Viktoria School Berlin‐Steglitz; Königin‐Luise School	Pupil	Born on November 9, 1914 and raised in Berlin‐Steglitz/Friedenau
1936–1941	Berlin, Germany Residing in Florastraße, 15	University of Berlin	Medical Student/Doctoral Candidate	Completion of medical studies (March 19, 1941) and conferral of doctorate (November 17, 1941) under the supervision of pharmacologist Wolfgang Heubner
1941 (May–Sept.)	Berlin, Ebenhausen, Bavaria, Germany	Bethanien Hospital Berlin (May 1 to July 15, 1941) ended due to “conscription of the director into the military” Sanatorium Ebenhausen (July 15 to September 30, 1941) as “holiday replacement”	Mandatory Assistant	Brief mandatory clinical rotations (StAH, 221–11, M. 2601)
1941–1943	Berlin, Germany Residing in Südendstraße 15	Institute of Anatomy (Prof. Hermann Stieve)	Assistant	Employment in anatomy; **voluntary resignation** on March 31, 1943. Documented as the only anatomist to leave the field during the Nazi era due to conscientious objection regarding the dissection of regime victims (specifically the *Red Orchestra* resistance group)
1943–1945	Berlin/Karlsbad (CZ)	State Police Hospital	Contracted Physician (Surgery)	Management of the surgical outpatient clinic; **active participation in the Albrecht Tietze resistance group**. Provided medical protection for politically persecuted individuals and those involved in the July 20 plot
1945–1947	Hamburg	Harbor Hospital Hamburg Finkenau Women's Hospital	Assistant Physician	Surgical work under Henning Brütt. Successful denazification (classified as exonerated due to resistance activities). She also worked as a volunteer assistant in gynecology and obstetrics from November 1, 1946 to December 31, 1946, likely at the Finkenau Women's Hospital, as documented in her medical professional records in Berlin (LAB, B Rep. 012 Nr. 9172)
1947–1949	Erlangen	Institute of Anatomy (FAU Erlangen‐Nürnberg)	Research Assistant/**De Facto Director**	Appointed assistant upon recommendation of Stieve and Heubner. Assumed de facto executive management of the institute (Dec 1947–1948) during the absence of acting director K. F. Bauer. **Likely the first female to hold such operational leadership in German anatomy**. Encountered significant gender‐based hostility and intrigue
1949–1950	Addis Ababa, Ethiopia	Haile Selassie Hospital	Probably Surgeon	**Commencement of a restless international career path**
1950–1951	Vellore, India	Medical College	Histology Instructor	Brief return to anatomical teaching in an international context
1951–1954	Würzburg, Germany	Institute of Pathology	Pathologist	Professional reorientation toward **pathology**. Conversion to Catholicism, which fundamentally shaped her later moral worldview
1955–1960	Canada (Oshawa)/USA	Oshawa General Hospital (Ontario)/Unknown US institutions, possibly Warren State Hospital	Pathologist/Researcher	Research on Wernicke's encephalopathy in Canada. Residence in the US (approx. 1958–1960)
1961–1963	Landstuhl, Germany	US Army Hospital	Civilian Pathologist	**Return to Germany:** Employment within the US military medical infrastructure, assisting William W. McLendon
~1965–?	Berlin	Auguste‐Viktoria Hospital	Pathologist	Late‐career return to her city of birth
~1972–197?	Munich/Eastern Bavaria	Red Cross Hospital	Pathologist	Documented employment in pathology shortly before retirement
1977–1988	London (UK)/Basel (Switzerland)	Private Residence	Retired/Independent Scholar	Drafting of a manuscript on Maupertuis and her own biography. Extensive correspondence with historian Golo Mann regarding the “ongoing war among Germans” and collective guilt
1988–2004	Munich/Eggstätt	Nursing Homes	Retired	Return to Bavaria. Death on April 23, 2004; her body was bequeathed to the Institute of Anatomy in Munich, closing the circle to her early career

Both she and subsequent researchers have attributed her resignation to her experience regarding the dissection of bodies of resistance fighters belonging to the *Rote Kapelle* group (Red Orchestra)—including Libertas Schulze‐Boysen (1913–1942)—who were executed on 22 December 1942. Crucially, some of these victims were personally known to Charlotte Pommer.^3^ ^(p10)^ Her resignation is cited as “the only known case of refusal to work by [a physician] employed in anatomy during the Nazi era”.^3^ ^(p10)^

Afterwards, she was assigned as a contracted police doctor in the surgical department of the State Hospital in Berlin under surgeon Professor Otto Hoche (1898–?) from April 1, 1943 to May 31, 1945. On May 31, 1945, the director of the State Hospital, Gustav Döderlein (1893–1980), issued her a certificate stating that “as a result of her diligence and skill, she has advanced her surgical training to such an extent that she can be entrusted with the independent management of the surgical outpatient clinic. Her surgical training is so advanced that she has already performed major surgical procedures herself” (BArch, R19/6451).

Her employment history in the later years of WWII, however, was more complex than previously stated. Due to diphtheria, she did not commence work until a month later, on May 1, 1943. Furthermore, on September 21, 1943, she was transferred to the police field hospital in Karlsbad/Czech Republic after the hospital in Berlin was bombed. She requested to leave the field hospital in February 1944. Although initially denied, she was eventually put in charge of the surgical outpatient clinic back at the Berlin State Hospital on May 15, 1944 (BArch, R19/6451). For her service during the relocation of the hospital to the military field hospital, she was awarded the War Merit Cross 2nd Class with Swords on May 11, 1944 (BArch, R19/6451 and Orth^3^ ^(p33)^).

During her tenure as head of the surgical outpatient clinic, Pommer was an active member of a resistance group centered around the physician Albrecht Tietze, which, according to Tietze himself, belonged to the circle of politician Wolf‐Heinrich von Helldorff (1896–1944).^12^ ^(p85)^ According to the contemporary witness Rudolf Wunderlich (1912–1988), a politician of the Communist Party of Germany and anti‐fascist who lived in hiding in Berlin and Leipzig from June 10, 1944 onwards after escaping from the Lichterfelde concentration camp, Tietze's group also maintained contact with journalist Theodor Haubach (1896–1945). He was part of what was later called the *Kreisauer Kreis*, a civilian resistance group of prominent Germans within the Third Reich that developed a theoretical framework for the political and social reconstruction of Germany based on Christian and humanistic values in anticipation of the Nazi regime's collapse (Reynolds^12^ ^(p132)^ and Behrens^19^ ^(p128)^).

While these connections require further research, they indicate that Tietze's group had close ties to individuals directly or indirectly involved in the July 20, 1944 plot, the failed attempt to assassinate Hitler and overthrow the Nazi regime led by *Wehrmacht* officers around Claus von Stauffenberg (1907–1944). According to Tietze's statement (quoted from his denazification files in Reynolds^12^ ^(p85f.)^), the members of his group included, among others, Greta Schellworth (1898–1968), pediatrician and head of the children's outpatient clinic at the State Police Hospital, who also maintained close contact with Theodor Haubach, Charlotte Pommer, and the head nurse of the internal medicine department of the State Police Hospital, Anna Buesse. In addition, Elisabeth Wengler, Tietze's housekeeper, as well as Edwin Kukula (1910–1991), a dermatologist at the State Police Hospital, and several police lieutenants were named by Tietze as members of the group. According to Pommer's report in Orth^3^ a larger circle of people was aware of the resistance activities.

The resistance group tried to keep Nazi opponents who had been admitted to hospital there for as long as possible. The duration of illnesses was prolonged on paper, and documents falsified in order to protect patients from abuse by the Gestapo and delay death sentences, as, for example, in the case of Wilhelm Roloff (1900–1979), who was privy to the assassination attempt on Adolf Hitler on 20 July 1944.^20^ ^(p131)^ The resistance actions of the group are described in greater detail in Orth^3^ and Reynolds.^12^

**FIGURE 1:**
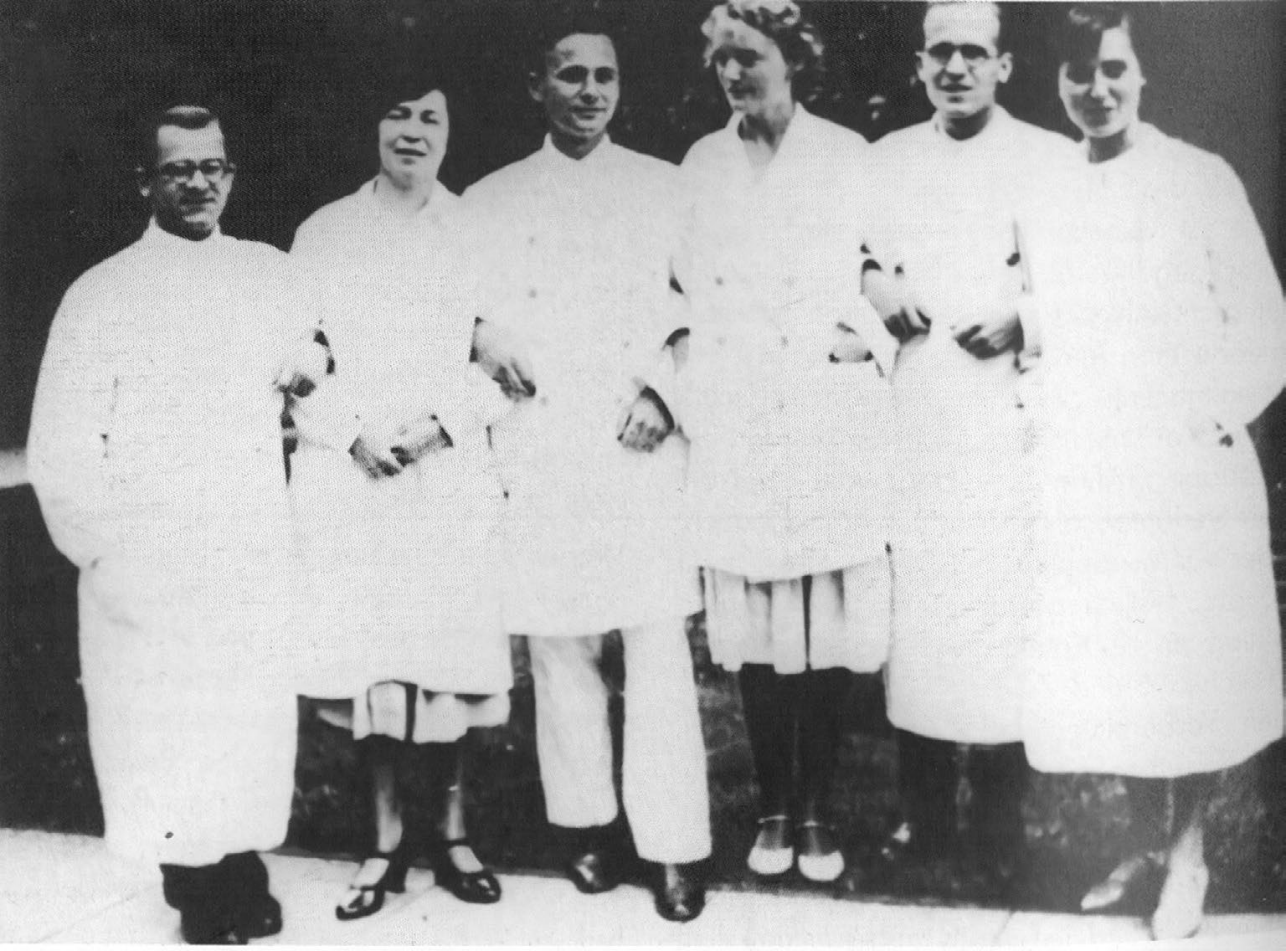
Albrecht Tietze (third from left) and his colleagues, probably from the State Police Hospital. First published in Meyer^21^ ^(p212)^; original from the personal archive of Albrecht Tietze. Photographer and current archive owner could not be determined.

Only a few group photographs of physicians from the State Police Hospital have been published to date or could be identified within the scope of this research. Among these, one photograph is of particular interest: a group photograph of six doctors—presumably from the State Police Hospital—dating from this period, which depicts Albrecht Tietze alongside colleagues circa 1944 (Meyer^21^ ^(p212)^, here Figure 1). The third individual from the left is identified as Albrecht Tietze. The photo was first published in 1999 by Winfried Meyer in his book “Verschwörer im KZ: Hans von Dohnanyi und die Häftlinge des 20. Juli 1944 im KZ Sachsenhausen” (Conspirators in the Concentration Camp: Hans von Dohnanyi and the Prisoners of July 20, 1944 in Sachsenhausen Concentration Camp). The photo comes from the former private archive of Wilhelm Tietze, Albrecht Tietze's late son. No information is available about the identities of the other individuals. Attempts to identify them have so far been unsuccessful. It remains unclear whether Charlotte Pommer is depicted in the photograph.

On March 10, 1945, Charlotte Pommer was arrested and interrogated by the Gestapo after investigations revealed that she, Alexandra Roloff (1910–1968), Wilhelm Roloff's wife at the time and Pommer's very close friend, and others were assisting politician and resistance fighter Hans Bernd Gisevius (1904–1974).^3^ ^(p11)^ Pommer was imprisoned in the transit prison on *Große Hamburger Straße*. She eventually became a secretary there, a position that—amidst the chaos of the war's end and through her contacts—enabled her to forge release papers for herself, her friend Alexandra, and two other supporters of Gisevius. Pommer and Alexandra Roloff escaped on April 21, 1945, and Charlotte Pommer returned to the State Hospital.^3^ ^(p12)^ She resigned from the hospital on May 31, 1945. Shortly thereafter, on July 12, 1945, her mother died of breast cancer at the State Police Hospital. Pommer was present in the hospital when her mother died, although she was no longer officially employed there (according to her mother's death certificate in LAB).

### The post‐war period: De facto leadership at the University of Erlangen (1945–1949)

From 1945 to 1947, Pommer settled in Hamburg, working with the surgeon Henning Brütt (1888–1979) at the Harbor Hospital Hamburg.^3^ ^(p58)^ On October 31, 1945, she received a questionnaire as part of the denazification process in the German occupation zones that all employees had to undergo. At that time, she resided in Hamburg‐Lokstedt (StAH, 221‐11, 621). In her questionnaire (StAH, 221‐11, M. 2601), she stated she had never been a member of any Nazi organizations or associations; she had merely been a member of the *Deutsche Studentenschaft* (German Student Union) between 1936 and 1940 and the *DRK* (German Red Cross) between 1936 and 1938. She detailed her detention by the Gestapo, citing Wilhelm and Alexandra Roloff as witnesses, and listed the reason for her arrest as: “For aiding political prisoners in the state prison and for ‘harbouring’ a political refugee from the Gestapo.” The military government in Hamburg subsequently approved Pommer on November 17, 1945 to work as a “hospital doctor” at the Harbor Hospital. This decision was confirmed again in April 1948 in a denazification proceeding in Erlangen (LAB, C Rep. 031‐02‐12 Nr. 81). Additionally, statements of neighbors were summarized there, according to which Pommer “lived her profession, was very reserved and always helpful.” This was also confirmed in these files by her parents' former caretaker, who knew the family well.

After 2 years in Hamburg (see Table 1), Charlotte Pommer moved to Erlangen to accept a post to work at the Institute of Anatomy, supported by letters of recommendation from Wolfgang Heubner and Hermann Stieve (UAE F2/1+2 No. 2611‐01626; Orth^3^ ^(p7)^). Although she had voluntarily resigned from Stieve's institute in Berlin, she received a laudatory recommendation from him after the war. Consistent with this, her denazification questionnaire listed the desire “to work in surgery” as the sole reason for leaving the Berlin Institute of Anatomy (see StAH, 221‐11, M. 2601). However, such administrative documents rarely served as venues for revealing inner moral conflicts; it is plausible that she chose a pragmatic explanation for official records. Regarding her motivation, Winkelmann^4^ suggests that it was the personal recognition of the victims—specifically the bodies of resistance fighters—that made it impossible for her to maintain the necessary professional detachment. The fact remains that Pommer is the only anatomist documented to have ceased her work in the field after encountering victims of the regime on the dissection table. While Stieve's positive recommendation might suggest the absence of personal animosity, it should be interpreted with caution. Given the political turmoil Stieve himself faced at the war's end—including accusations from his own students and critical reports in the foreign press—he might have had strategic reasons to support an acknowledged resistance fighter such as Pommer, regardless of his personal feelings toward her.

However, complications arose regarding a further recommendation for Pommer, as evidenced by a letter dated November 7, 1947 in Stieve's estate (HU UA, NL Stieve, 115). There were apparently considerations for Pommer to reapply to Stieve in Berlin, possibly due to an opening becoming available in Stieve's department following the dismissal or transfer of Auguste Hoffmann by the military government. Hoffmann, one of the first female professors in sports medicine, had conducted racial research on the German Olympic team in 1936 and worked for Stieve after the war until her transfer to Greifswald in 1948 for political reasons, where she briefly headed the Institute of Anatomy.^22^ By this point, Pommer was already in Erlangen. Heubner wrote to Stieve regarding the matter:

After our [Stieve and Heubner] conversation, I will not recommend Dr Charlotte Pommer to apply to you. From your remarks, I regret to conclude that the mere fact that I am on friendly terms with Miss Pommer arouses your mistrust of her. However, I hold Miss Pommer in too high a regard as a person to expose her to such mistrust.

Despite this rejection, Stieve provided another excellent reference much later on 23 February 1952, describing Pommer's work as follows: “She participated primarily in teaching the histology course and dissection exercises, as well as in presentations. She fulfilled all her duties in the best possible manner and possesses a special talent for teaching. Her conduct has given no cause for complaint whatsoever” (LAB, B Rep. 012 Nr. 9172).

Wolfgang Heubner was not only her doctoral supervisor^3^ ^(p73)^ but also a friend and, as revealed in letters to Golo Mann, a role model for Pommer. It is therefore notable that Pommer was not mentioned in Heubner's very detailed diaries until 3 August 1947, when she visited his house for coffee (ADGPT, TBH 19). In her manuscript (in Orth^3^ ^(p78f.)^, written in the 1960s and 70s), she describes a meeting in the spring of 1944 to manufacture a “suicide pill” for Werner von Alvensleben (1875–1947). Heubner may have omitted this meeting and his acquaintance with her from his wartime diaries to protect both himself and her, likely aware of her resistance activities, given that he produced the “suicide pill” himself. Pommer noted in her manuscript that keeping diaries during the Second World War was potentially very dangerous, which may explain Heubner's silence.^3^ It appears undisputed that Heubner created the “suicide pill,” as Werner von Alvensleben himself told his daughter Annali von Alvensleben about it.^11^ ^(p110)^

Two days later, on August 5, 1947, Pommer appears again in the diary, having invited the Heubner and Tietze families to her home and that of “her friend (original: *ihre Freundin*) Mrs Siller in Steglitz, Südendstr. 15.” Heubner notes that “Mrs General [Lina] Lindemann was also present” (ADGPT, TBH 19). She was the widow of General Fritz Lindemann (1894–1944), who participated in the July 20 plot and was severely wounded during his arrest. He passed away in the State Police Hospital under the medical care of, among others, Tietze and Pommer.^3^ The friend, Hertha Siller, was a respiratory therapist in Berlin; the precise nature of her relationship with Pommer remains unclear. According to the registration card register (B Rep. 021 in the State Archive Berlin), Pommer lived there until March 3, 1947. In 1952, while working in Würzburg, Pommer still listed Südendstraße 15 and Hertha Siller as the contact for a registered letter from the Senator for Health concerning her work qualification (LAB, B Rep. 012 Nr. 9172).

On August 26, 1947, Heubner reported that Charlotte Pommer was “on her way to Erlangen – as an anatomist!” (ADGPT, TBH 19). At the time Pommer took up her position, she was residing in St. Pauli/Hamburg (ADGPT, TBH 19). On August 27, 1947, she met Heubner again. He also recorded the dispute with Stieve regarding Auguste Hoffmann's dismissal and the suggestion concerning Pommer, as stated above in his diary. He wrote:

7 November [1947] After lectures and a meeting with the dean at Stieve's office, a rather heated argument ensued, as he accused me of being responsible for Ms Hoffmann's dismissal and viewed my suggestion regarding Miss Charlotte Pommer with great suspicion. (ADGPT, TBH 19)

Heubner and Stieve apparently had long‐standing disputes. A diary entry from 4 February 1951 describes Stieve refusing to shake Heubner's hand (ADGPT, TBH 22). These tensions probably stemmed from political reasons, as Heubner considered Stieve to be politically incriminated and expressed this to the dean of the medical faculty in Berlin. Charlotte Pommer's name appears only once more in Heubner's diaries, on October 28, 1953, when he spoke to a mutual friend of Tietze and Pommer, the police doctor Dr. Willy Veit (1912–?) (ADGPT, TBH 23).

At that time, the Institute of Anatomy in Erlangen was under the interim direction of Karl Friedrich Bauer (1901–1985). Bauer frequently traveled to Switzerland on research trips with the physician Paul Niehans (1882–1971), who was best known as the developer of “*Frischzellentherapie*” (fresh cell therapy), in which Bauer also participated. Fresh cell therapy is a controversial practice in which fresh animal fetal cells are injected to supposedly revitalize human tissue. Although Niehans's methods are widely regarded as pseudoscientific by modern medical standards, he found a significant following among the global elite in the mid‐20th century.

Due to his planned absence, Bauer himself proposed on December 27, 1947 that Pommer take over the “management of the Institute of Anatomy (*Geschäftsführung der Anatomischen Anstalt*) […] until the final appointment of an institute director” (UAE F2/1 No. 2006a, p. 24). The dean approved this proposal, recommended this course of action to the Ministry of Education, and even expressed a wish to transfer the management to Pommer permanently if Bauer's provisional direction expired (UAE F2/1 No. 2006a, p. 25). Pommer had been hired only shortly before, on August 15, 1947, with “the best recommendations from the Director of Berlin Anatomy Prof. Stieve and Director of the Pharmacological Institute of the University of Berlin Prof. Dr W. Heubner” (UAE F2/1+2 No. 2611‐01626). She was officially appointed as a permanent research assistant at the Erlangen Institute of Anatomy and entrusted with the deputy duties of a prosector (UAE F2/1+2 Nr. 2611‐01626).

Ultimately, Pommer did not return to Berlin but took over the management of the institute in Erlangen during Bauer's absence in late 1947, when she was just 32 years old. In September 1948, an application was submitted to the Bavarian Ministry of Culture, asking for additional remuneration for Pommer for her interim management of the institute serving as administrative proof of her status (BayHStA, MK 72027). While it is doubtful she ever held the official “*de jure*” status of acting director conferred by the Free State of Bavaria, other textual evidence sufficiently substantiates this “de facto” position. Josef Beck (1891–1966), the dean of the medical faculty in Erlangen, wrote on July 7, 1948 (UAE C3/1 No. 283):

Miss Dr Charlotte Pommer, born on 9 November 1914, has been employed at the Institute of Anatomy of the University of Erlangen since 15 August 1947. She was entrusted with the preparations for the histology and dissection course for 650 students, which means that she prepared many thousands of specimens herself. She carried out her teaching duties on selected chapters of macroscopic and microscopic anatomy with great diligence and to the utmost satisfaction. She was mainly involved in teaching and was very popular with the students. From 13 December 1947 until the end of the winter semester of 1947/48, she ran the institute completely independently, taking over the director's teaching duties and examinations. During this time in particular, she proved that she was up to the task and was recognised and respected by her subordinates. Her leadership was always impeccable. Dr Pommer intends to leave the University of Erlangen to go to Addis Ababa.

**FIGURE 2:**
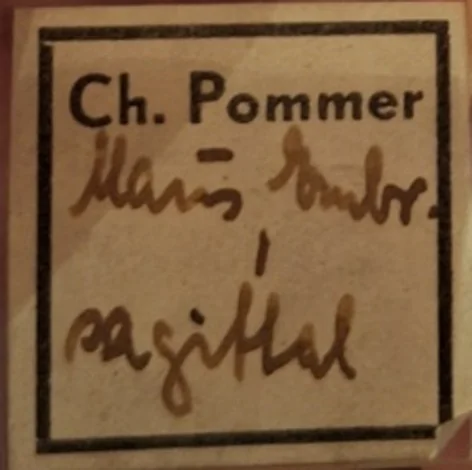
Label of a histological specimen of a mouse embryo, featuring the inscription of the preparer “Ch. Pommer.” Photo: T.S. Goldmann.

This dean's assessment is corroborated by contemporaneous personnel actions: Pommer dismissed a laborer, Karl Will, on January 15, 1948 for theft of institute property, while she requested leniency to avoid reporting him to the authorities due to his history as a concentration camp prisoner (UAE A6/3 Nr. 57). Physical evidence in the form of histological specimens can still be identified today at the Erlangen Institute of Anatomy, further illustrating Pommer's work there (Figure 2). These items together document operational responsibilities (teaching, examinations, personnel) consistent with de facto leadership.

Additionally, she had to contend with her difficult colleague Johannes Hett (1894–1986), who described her in a negative tone in a statement dated August 4, 1948 (UAE F2/1 Nr. 2296b). During the Nazi era, Hett had been director of the Histology Department from 1936 onwards and, after the war, briefly served as acting director of the institute until he was dismissed by the military government and had to leave his position, while still living in the institute with his family. He regained his teaching license in 1948. Hett, who had conflicts with many faculty members—including Albert Hasselwander (1877–1954), the former director of the Institute from 1918–1945, and Karl‐Friedrich Bauer—claimed Pommer had incited discord against him, eavesdropped on a telephone conversation, and reported it to the dean in a “distorted manner.” He also alleged she confiscated his share of the institute garden. This conflict highlights the lack of a supportive professional network for Pommer. In post‐war Erlangen, she found herself navigating a thicket of (political) intrigue and deep interpersonal conflict. The institute was populated by difficult characters such as Johannes Hett and Otto Popp (1897–1961), who struggled to integrate into new post‐war structures, both former pupils of Hermann Stieve in Halle (Hett) and Berlin (Popp). The internal conflicts within the Erlangen Institute of Anatomy during the later years of WWII and the post‐war period have, as of today, scarcely been investigated. Projects are currently underway to examine these events. First information regarding Popp and Hett can be found in Hildebrandt^7^ ^(p259)^ Unlike her male colleagues, Pommer could not rely on an established network or academic mentors to shield her from these internal power struggles, also given that her main advocate, Bauer, was absent.

Hett stated that Pommer “continues to head the department – vacated by my dismissal – as an unqualified (non habilitated; lacking the formal postdoctoral qualification for independent research and teaching) person, holds the physics exam at Easter as the only “subject representative” and gives lectures on behalf of the faculty” (UAE F2/1 Nr. 2296b, p. 72). He also notes that Pommer spoke with the Ministry of Culture in Munich and visited the retired Albert Hasselwander in Arlaching, Bavaria and suspects that he was also denounced there (UAE F2/1 No. 2296b, p. 92). This conflict with Hett, and another with Friedrich Büthe (1917–?), an assistant at the institute—who suspected that the reasons for his dismissal were based on statements made by Pommer to Bauer (see UAE F2/1+2 Nr. 2612‐01430)—eventually reached the dean's office but was settled when Pommer left the university (UAE B2.3.1). While Hett was generally known for his conflict‐prone nature, it is likely that he—and other male colleagues—perceived the younger, in their opinion “unqualified” woman as an “easy target” within the male‐dominated academic hierarchy, making it even harder for Pommer.

All these previous findings identify Charlotte Pommer as possibly the first—and certainly one of the first de facto—female directors of an Institute of Anatomy in Germany, and likely the first woman to hold such a management position at FAU. No other examples prior to 1947 have been identified for Erlangen and Germany (for an overview for Erlangen, see Wittern‐Sterzel^23^). The first woman to become at least interim director of an anatomical institute in Germany was probably Auguste Hoffmann, who, as already described, was transferred for political reasons and officially (unlike Pommer) became interim director of the Anatomical Institute in Greifswald in 1948. The first woman to become a full professor and chair holder for anatomy in Germany was Anne Lise Schubel (1907–1988), initially on an interim basis at the University of Rostock in 1957, then permanently at the University of Göttingen from 1 January 1958.^24^ A little earlier, in 1953, Emmi Hagen (1918–1968) became at least head of the Department of Experimental Biology at the Anatomical Institute of the University of Bonn, before becoming a full professor there in 1967.^25^

### A restless career: International stations and pathology (1949–1978)

As summarized in Table 1, Pommer finally left Erlangen in 1949 to embark on a restless international career path that took her to Ethiopia and India before she returned to Germany in 1951 to work in Würzburg as a pathologist (UAE F2/1+2 No. 2611‐01626). Information from the US Embassy in Ethiopia supports this, reporting on “German Infiltration into Ethiopia” in June 1950. The document mentions Wilhelm Roloff—hinting at his potential involvement with British intelligence—and Charlotte Pommer at the Haile Selassie Hospital (NA Bigelow to Department of State; 762.5275/6‐2350; DF 1910‐1963; General Records of Department of State; RG 53; NACP). During her subsequent tenure at the Institute of Pathology in Würzburg, she converted to Catholicism—a step that would fundamentally shape her later moral worldview as described below.^3^ ^(p110)^

Afterwards, she moved to Canada to work at the Oshawa General Hospital/Ontario. Her presence in Canada by 1954 is evidenced by correspondence from Oshawa addressed to the biochemist and Nobel laureate Feodor Lynen (1911–1979), in which she mentions attending his lecture in Würzburg in the spring of “that year,” probably meaning 1954. Notably, following her departure from Erlangen, she primarily identified herself as a pathologist rather than an anatomist (AMPG, III/31B/60 No. 66).

In Oshawa, Pommer appears to have conducted research on Wernicke's encephalopathy (AMPG, III/31B/60 Nr. 66). In a letter to Golo Mann dated April 8, 1989, she mentioned that she was a specialist in pathology (SLA‐GM, B‐4‐j‐9.8). Interestingly, her relocation to Canada occurred shortly after Wilhelm Roloff, Alexandra Roloff's now ex‐husband and former protégé of Pommer and Tietze at the State Police Hospital, emigrated there in 1953, paralleling her earlier engagement in Ethiopia. Although exact dates remain unconfirmed, she was certainly residing in Canada by January 1955, as demonstrated by a letter to Alexandra Roloff (then Weber) dated January 3, 1955. In this correspondence, she described applying for a US visa at the behest of Philipp Schwartz (1894–1977)—a pathologist who had previously held professorships in Frankfurt and, following his dismissal by the Nazis, in Istanbul—who had invited her to the Warren State Hospital/Pennsylvania for “anatomical studies” (NA Pommer to Department of Foreign Affairs; 116.31/1–355; DF 1910–1963; General Records of Department of State; RG 53; NACP).

She sought Alexandra's advice on expediting the visa process, remarking with humor: “So, I want to go back behind bars, but this time with a house key.” Pommer ironically compares her new medical position in a closed psychiatric ward at Warren State Hospital to the time she spent in prison with Alexandra Roloff, noting that although she is returning “behind bars,” she now has the “house key” and no longer lives in captivity like a prisoner. Pommer also mentioned Maria Daelen (1905–1993), with whom Pommer had evidently maintained correspondence for a period. She was a former Berlin physician active in the resistance during the Nazi era, friend of Pommer and German government official who later became a leading figure in the health department of the West German Ministry of the Interior. This letter, however, never reached Alexandra, as the US postal service was unable to locate her. It remains unknown whether Pommer eventually worked with Schwartz. She reported residing in the United States from at least 1958 to 1960, likely employed at a Catholic hospital administered by German nuns.^3^ ^(p9)^ Although an A‐File number was located, Charlotte Pommer's visa documents could not be found in the US archives.

**FIGURE 3:**
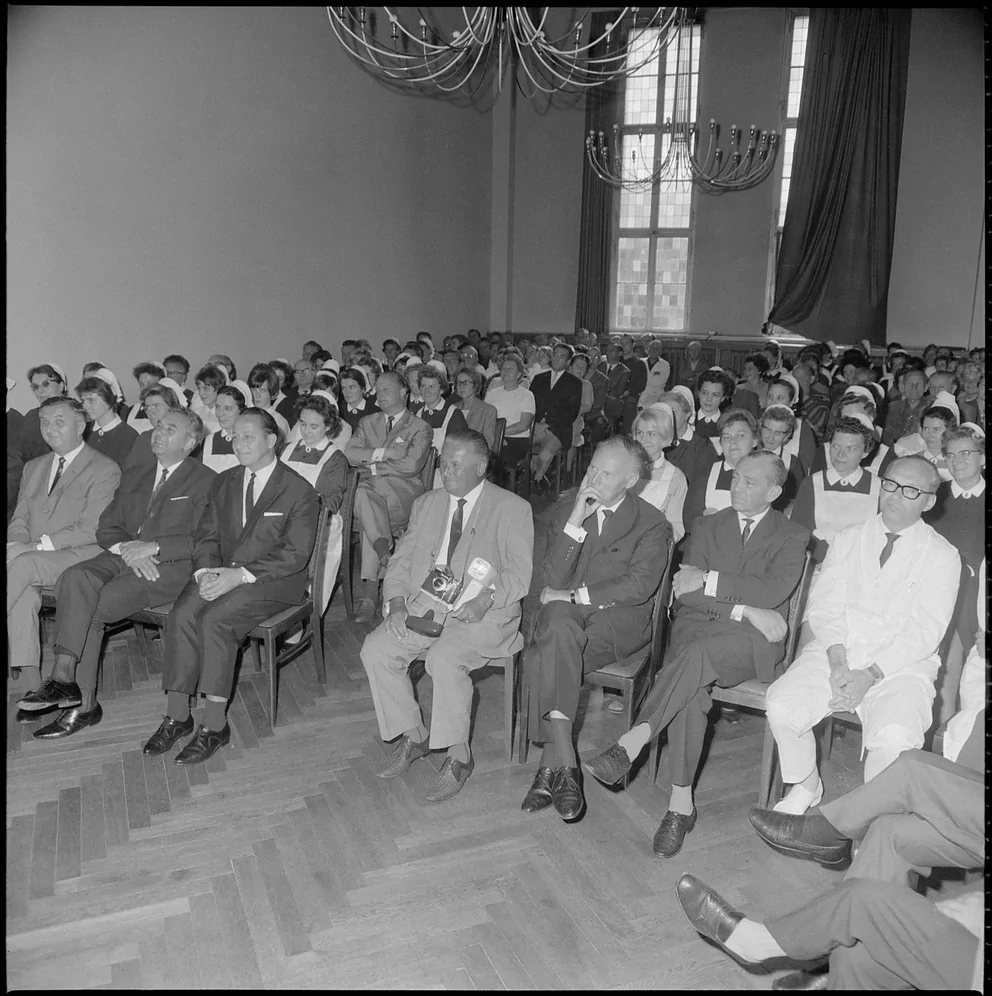
Photograph of the Ceremony marking the 60th anniversary of the Auguste‐Viktoria Hospital in 1966; third from left: Haase (district councillor); from right to left: Prof. Dr. Brandenburg; Prof. Dr. Maatz; Prof. Dr. Pfeffer. Landesarchiv Berlin, F Rep. 290 (04) Nr. 0115396 / Photographer: Ludwig Ehlers.

By 1960 she had returned to Germany, describing a meeting with Jürg Zutt (1893–1980), a psychiatrist and neurologist in Frankfurt and former attending physician of Christine von Dohnanyi (1903–1965), who was active in the resistance.^3^ ^(p73)^ Subsequently, she was employed as a civilian pathologist at the US Army Hospital in Landstuhl between 1961 and 1963, assisting William W. McLendon (1930–2024) during his travels through Europe.^26^ ^(p32)^ According to Pommer's former assistant, Udo Krings, she worked as a pathologist at the Auguste‐Viktoria Hospital in Berlin‐Schöneberg in 1965/66, where he conducted his doctoral research.^27^ However, the exact timeframe of her work there is unknown. A photograph from the hospital's 60th anniversary on October 4, 1966 may depict her, as the head of pathology Wolfgang Brandenburg is also present (see Figure 3). By 1972, she was employed as a pathologist in Eastern Bavaria, possibly in Munich, and by 1974 she was evidently working at the Red Cross Hospital in Munich (SLA‐GM, B‐4‐j‐8.9; Orth^3^ ^(p9)^; AMPG III/31B/60 No. 66).

### Retirement and historical research: The Maupertuis manuscript (1978–2004)

By the end of 1977, Pommer had retired or departed from her position at the Red Cross Hospital. Correspondence with the Central Office of the State Justice Administrations for the Investigation of National Socialist Crimes dated December 14, 1977 and Golo Mann dated August 12, 1978 places her at 14 Exeter Road, London/UK (BArch, B 162/31092; SLA‐GM, B‐4‐c‐5.1). Although the earliest version of her autobiographical manuscript probably dates from the 1960s, the correspondence with the Central Office of the State Justice Administrations for the Investigation of National Socialist Crimes (BArch, B 162/31092) indicates that she was still actively working on the text during the 1970s in London. Subsequently, she moved between Basel/Switzerland and London (see Table 1). The Basel City Archives record her registration in Basel from April 19, 1970 to July 17, 1980—though evidently not as a permanent resident—before she officially returned to London (according to the City of Basel Residents' Registration Office). She appears to have resided in Basel again between at least December 8, 1986 and April 15, 1988, prior to returning to Germany.

During this period, she also worked on a biography of Pierre Louis Moreau de Maupertuis (1698–1759), a pioneer in heredity research. She mentioned this project in a letter to Golo Mann and apparently sent him the work, although the draft was not found in his estate (SLA‐GM, B‐4‐j‐8.9; SLA‐GM, B‐4‐j‐9.8). However, a manuscript by Pommer was located in the library of the *Stiftung Preußische Schlösser und Gärten Berlin‐Brandenburg* (Prussian Palaces and Gardens Foundation Berlin‐Brandenburg) under the reference number Q‐P‐004 (cited here as Pommer,^28^). In this text, she undertakes an art‐historical analysis of a portrait of Maupertuis.

**FIGURE 4:**
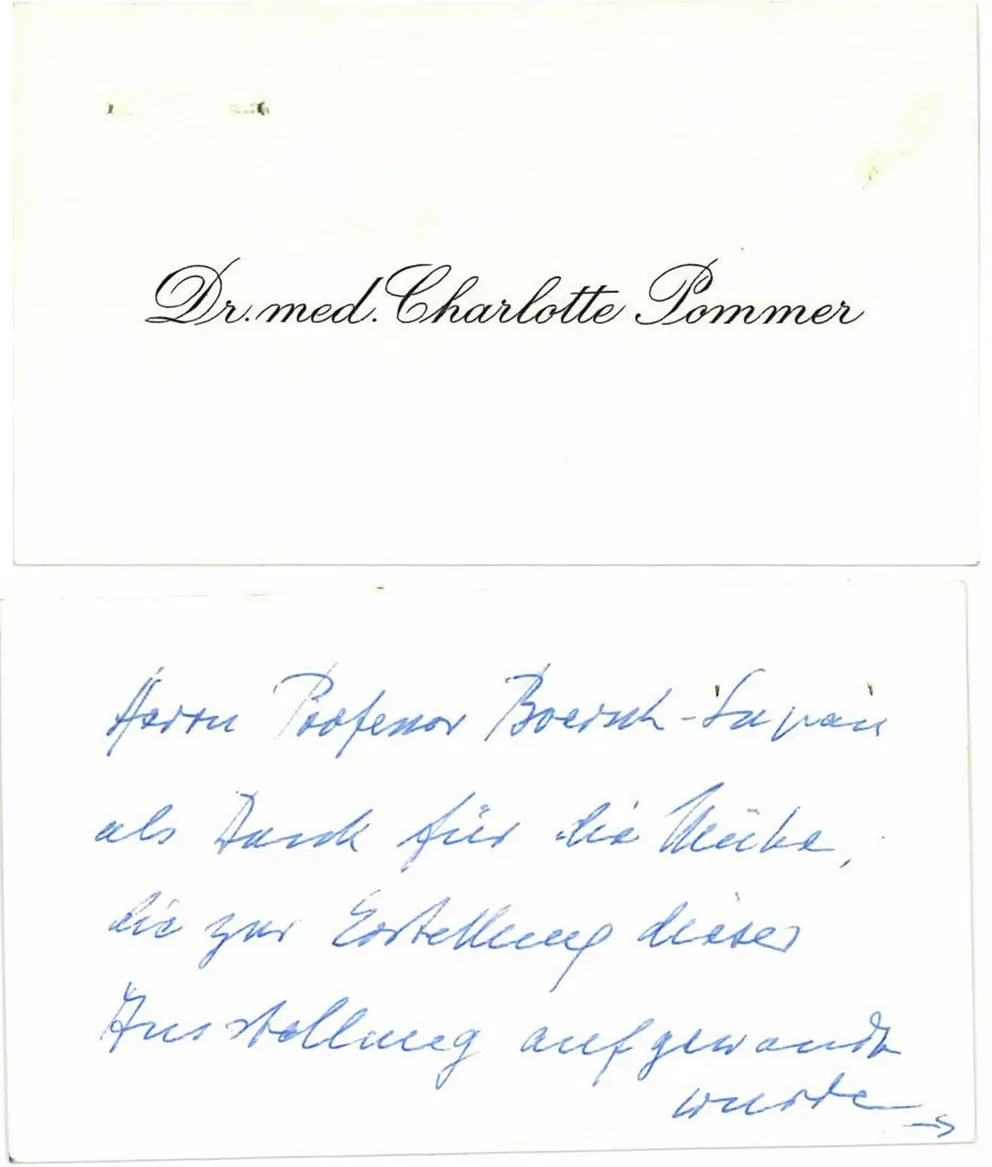
A business card of Charlotte Pommer, bearing the printed name “Dr. Charlotte Pommer,” which is attached to her manuscript. On the Reverse of the business card, it bears the following handwritten inscription: “To Professor Börsch‐Supan, in gratitude for the effort expended in the preparation of this exhibition ‐>”Copy of Q‐P‐004.

It is probable that she donated this manuscript in conjunction with an exhibition on Friedrich II of Prussia, curated by Helmut Börsch‐Supan, a German art historian, in 1986 for the *Geheimes Staatsarchiv Preußischer Kulturbesitz* (Prussian Cultural Heritage Foundation) (see Figure 4). It remains unclear whether the manuscript^28^ is identical to the one sent to Mann or merely an excerpt regarding the portrait. Nevertheless, it represents the only textual evidence of her research on Maupertuis and stands as the third surviving publication (alongside her dissertation and the manuscript in Orth^3^). This work is surrounded by controversy; Pommer claimed that “no West German is interested in my work on Maupertuis, but East Berlin and Leningrad are, and they have their agents steal my essays – purely amateur work” (SLA‐GM, B‐4‐j‐8.9). As no Stasi files relating to these accusations have been found, the veracity of her claims cannot be verified. In her letters, Charlotte Pommer repeatedly alleged persecution by “Communists,” yet there is no evidence to substantiate these fears.

## THE “ONGOING WAR AMONG GERMANS”: POMMER'S WARTIME REFLECTION, BELIEFS AND WORLDVIEW

To fully understand Charlotte Pommer's worldview, her reflections must be contextualized within her unique biographical situation: she was a woman in a male‐dominated discipline devoid of female leadership models, and a politically independent thinking individual who refused to “adapt” to the regime—unlike contemporaries like the anatomists Wolfgang Bargmann (1906–1978) or Heinrich von Hayek (1900–1969).^29^ Lacking a supportive professional network, she found herself in the immediate post‐war period navigating the already mentioned climate of rivalries in the Erlangen Institute of Anatomy, characterized by political intrigue and conflict with difficult personalities such as Johannes Hett and Otto Popp. This professional and moral isolation forms the backdrop for her later correspondence.

Notably, Pommer's letters to Golo Mann employ phrasing that parallels her manuscript in Orth.^3^ In her initial letter to Mann in 1978, she discussed the war of “Germans against Germans”—a conflict she viewed as ongoing—using the exact terminology found in her earlier manuscript.^3^ ^(p118)^ She cited two contemporary circumstances as “proof” of this thesis, addressing highly political issues and her personal processing of the war:

First, Pommer described abortion as an evil, without ever explicitly naming the procedure (see Orth^3^ ^(p117f.)^). This is consistent with her apparently very deep Catholic convictions following her conversion in the 1950s and stands in opposition to the reform of Section 218 of the German Criminal Code in the 1970s and the social changes driven by the 1968 protests (see Behren^30^). For Pommer, this likely represented one part of this war “Germans against Germans.”

Second, in her first letter to Golo Mann of August 12, 1978, she referred directly to an interview Mann gave to the newspaper *Bild am Sonntag* on August 6, 1978. The following correspondence with Golo Mann only came about because she responded to this article in the manner of a “letter to the editor.” Mann replied to two of her letters (dated December 8, 1986 and spring 1987). In the article, Mann proposed a general amnesty for Germany, whilst emphasizing that the German reputation was not unblemished: “There is a curse on the German name, but it will gradually fade away”.^31^ ^(p12)^ Pommer partially agreed with Mann regarding the potential for collective guilt, concurring that “it is a fate to be German, especially if one had to remain in Germany between 1933 and 1945 […] but the justified feeling, as a German, of belonging to this country, even if not to the government.” She added: “In my opinion, this is also how we should understand Furtwängler's decision to remain, whose performances were a great comfort to us.” She further mentioned her doctoral supervisor, Heubner, noting: “I do not want to erase from my life the role model that I received from my doctoral supervisor ‘in the stream of time’,” even though he is a German.

However, Pommer took offense at the distinction Mann drew between different types of resistance fighters. Mann argued for a clear differentiation between those who actively resisted, such as Stauffenberg, and those who “only clenched their fists in their pockets,” implying the latter shared in German guilt.^31^ ^(p12f.)^ Pommer confronted Mann directly:

You can reproach me for not having killed Adolf Hitler. But I will not allow myself to be imprisoned by your assumption of collective guilt. You can say that I have ‘come to nothing’ because my main work is still to suppress what I saw and experienced and had to bear under an excessive burden of responsibility at a young age. I refuse to be included in collective guilt by anyone, including you, who views Germany from a historical perspective and not from direct experience.

To substantiate her position, she noted that “Fabian Schlabrendorff […] already made this very clear to Pastor [Martin] Niemöller [in 1945].” These exchanges reveal her sentiments regarding the war and her role in it nearly 34 years after the events. Fabian von Schlabrendorff (1907–1980) was a jurist and officer in the military resistance who actively participated in assassination attempts against Hitler, while Martin Niemöller (1892–1984) was a prominent theologian of the Confessing Church. By invoking them, Pommer possibly aligned herself with active resistance while partially rejecting the retrospective judgment of those who had lived in exile. She summarized her view thus:

The battle between Germans and Germans has now been going on for 50 years. For me, the shooting of two captains [*Hauptleute*] on Magdeburger Platz marked the beginning of violence against the state, which continued from then on, from 1933 to 1945, in the opposite direction – with only a brief interruption under Konrad Adenauer. The tearing apart of Germans against Germans continues on several levels, which is highly undignified. (SLA‐GM, B‐4‐c‐5.1)

It is possible that Pommer is mistaken about the location and is referring to the murder of Paul Anlauf (1882–1931) and Franz Lenck (1892–1931) on Bülowplatz, Berlin (now Rosa‐Luxemburg‐Platz) on August 9, 1931. The victims were two police captains who were killed by members of the paramilitary wing of the Communist Party of Germany (KPD). This would also explain her remark “in the opposite direction,” since the extreme left‐wing KPD was responsible for the murders in 1931, while right‐wing extremists, Nazis, were active after 1933. All this must also be seen in connection with the militant left‐wing extremist Red Army Faction (RAF), which had been active since 1968 and was omnipresent at the time of Pommer's letter (1978), possibly representing an example of the ongoing “tearing apart of Germans against Germans.”

Her memories of the war and the events surrounding July 20, 1944 were important to her self‐conception, and she reacted strongly when she encountered what she considered to be inaccuracies about them in public discourse. This becomes clear in a second letter to Mann dated December 8, 1986. There, Charlotte Pommer asked Mann to use his influence to stop or edit the broadcast of an interview on RIAS Berlin with “Baron von Hammerstein.” It is unclear to which Baron von Hammerstein she referred, though it was possibly Ludwig Freiherr von Hammerstein‐Equord (1919–1996). Pommer described the interview as “an insult to the spirit of my patients who died in prison or after recovery at the hands of the executioner (if those can even be wounded/insulted)” (SLA‐GM, B‐4‐j‐8.9). Unfortunately, the content of this interview has not been reconstructed.

In her final letter to Golo Mann in 1989—the latest surviving textual record of Pommer—her stance on communism is again evident. Referring to a text by Mann in the *Schweizer Illustrierte* dated December 15, 1989,^32^ where he observed that “we see a large part of the Swiss press patiently encouraging the non‐communist world to adopt a policy of resistance and a combative stance, which, however, its own tradition strictly forbids it from participating in within the Swiss political system”,^32^ ^(p15)^ Charlotte Pommer responded with the clear call “Helvetia, wake up!” (SLA‐GM, B‐4‐j‐9.8). She also offered a rare remark on her parents, confirming “[her] parents' attitude” probably as bourgeois democratic and state‐loyal based on Mann's comments in his book *Erinnerungen und Gedanken* ^33^ regarding political figures such as Otto Braun (1872–1955), former Prime Minister of the Free State of Prussia (1920–1932) and a leading Social Democrat, or Carl Severing (1875–1952), a Social Democratic politician who served as Prussian Minister of the Interior (1920–1926, 1930–1932) and Reich Minister of the Interior (1928–1930) (SLA‐GM, B‐4‐j‐9.8). The only other thing that could be ascertained about her parents in a political context was that her father, Walter Pommer, joined the NSDAP on 1 March 1940, with membership number 7.547.041.

She concluded the letter with an excerpt from Walther von der Vogelweide's Middle High German poem *Ich saz uf eime steine*, which also appears in Orth^3^ ^(p118)^: “Disloyalty is in the ambush, violence is on the street, peace and justice are sorely wounded” (“Untrûve ist in der sâze, gewalt wart uff der strasse, fride un recht sind hare wund”), mourning the violation of peace and justice.

Interesting parallels can be drawn between her worldview and her restless post‐war trajectory. Unlike the Austrian anatomist Clara Zawitsch‐Ossenitz (1892–1956), who returned from exile as an active Catholic resister to successfully direct the Institute for Histology and Embryology in Graz, Pommer did not find a permanent institutional home. Returning to Germany on April 15, 1988, Pommer initially had no permanent residence, living in hotels (SLA‐GM, B‐4‐j‐9.8) before moving to nursing homes in Munich and Haar, Bavaria, by 1993, and finally to Eggstätt, Bavaria, in 1995. This restlessness—spanning continents from Ethiopia and India to North America—may be interpreted as a consequence of her wartime motivations or possibly as a professional structural barrier because of her gender. Having witnessed anatomical body procurement during National Socialism and participated in resistance, she was perhaps unable to settle in a society she viewed as morally compromised, a society that strengthened abortion rights and was exposed to German‐German conflicts, now, for example, through the RAF and a divided Germany. Perhaps she was seeking peace globally that she could not find locally. She died on 23 April 2004 and bequeathed her body to the Institute of Anatomy in Munich.^3^ ^(p10)^

## DISCUSSION: NEW INSIGHTS INTO CHARLOTTE POMMER'S LIFE AND WHAT THEY MEAN

Archival records demonstrate that Charlotte Pommer performed the essential duties of institute management in Erlangen from December 1947 until the end of the 1947/48 winter semester. The Dean's contemporary assessment highlights her organization and implementation of dissection and histology courses for large groups, as well as her independent conducting of examinations—facts supported by other witnesses (UAE C3/1 No. 283). Personnel files and appointment certificates confirm her operational duties as a research assistant and deputy prosector (UAE F2/1 No. 2611‐01626), while a request to the ministry for additional remuneration documents the institutional recognition of these duties (BayHStA, MK 72027). Furthermore, administrative records, such as the dismissal of employees and internal case processing, attest to her managerial authority (UAE A6/3 No. 57).

These consistent records justify describing Pommer as the “de facto*”* director, despite the absence of a formal “*de jure”* appointment. Methodological limitations persist, notably gaps in personnel files, sparse biographical traces, and limited contemporary witness statements. Comparative context is also limited: published scholarship on women in German anatomy is sparse. Recent studies highlight increasing attention to gender and institutional practice,^1^, ^2^ but no dedicated national survey of female managerial roles in anatomical institutes exists. Pommer's case, therefore, serves as a call for future comparative work.

To fully appreciate the significance of Pommer's unofficial directorship, it must be viewed within the broader historical context of female physicians in Germany—a history characterized by structural exclusion and delayed recognition. Until the late 19th century, eminent German anatomists vehemently opposed women entering medical school. Notably, Wilhelm von Waldeyer‐Hartz (1836–1921), the renowned director of the Berlin Institute of Anatomy, argued in a widely publicized speech in 1888 that women were mostly anatomically and intellectually unfit for medical study.^34^ Consequently, women were not admitted to medical studies in Germany until the turn of the century, starting in Baden in 1900 and finally in Prussia in 1908.

Prior to these reforms, pioneers such as Elisabeth Winterhalter (1856–1952) were forced to seek education abroad, particularly in Switzerland, where the University of Zurich had admitted women as early as 1864. Upon returning, these Swiss‐educated physicians often faced significant legal battles to have their degrees recognized in Germany. While the Italian physicist and anatomist Laura Bassi (1711–1778) had been appointed the first female salaried university chair in Bologna much earlier, the German‐speaking world lagged significantly behind. Hedwig Frey (1877–1938) at the University of Zurich eventually became the first female professor of anatomy in the German‐speaking area in 1924, albeit initially only as a Senior lecturer.^35^ In Germany, Rahel Hirsch (1870–1953) became the first woman to receive a medical professorship in Prussia at the Charité in 1913; however, she remained a Senior lecturer and never held a chair.^36^

Against this backdrop of women traditionally being denied institutional support and promotion, Pommer's de facto leadership in 1947 appears even more significant. It represents a continuation of a historical pattern where women in medicine performed the duties of leadership without receiving the commensurate official status or title. A nuanced interpretation—supported by Pommer's denazification questionnaire and contemporaneous testimonies—suggests that her resignation from Stieve's Berlin institute was dictated by a profound conscientious objection. This decision likely reflected the cumulative burden of the institute's practices, potentially catalyzed by the personal emotional impact of encountering bodies of executed persons she may have known, rather than solely a strategic career move.

Furthermore, these factors should not be viewed as mutually exclusive hypotheses. The archival evidence permitting readings of a career choice toward surgery or local personnel politics does not invalidate the moral dimension of her departure. Rather, Pommer's subsequent professional reorientation toward surgery and pathology is entirely consistent with a refusal to continue in anatomy under the prevailing conditions. Here, Pommer's trajectory contrasts sharply with contemporaries such as the already mentioned Wolfgang Bargmann or Heinrich von Hayek. While these male colleagues “adapted” to the political circumstances of the Nazi regime to further their academic careers, Pommer prioritized her moral convictions.^29^ Reacting to the reality of body procurement under the Nazi regime, she chose to leave the field of anatomy, pursuing surgical practice as a means to remain in medicine, probably without compromising her ethical standards.

Furthermore, her experiences must be viewed through the lens of gender. As a woman in a male‐dominated discipline, Pommer belonged to a small minority without female role models in leadership positions. Consequently, she likely faced not only political but also structural isolation, with her professional potential rarely receiving the serious consideration accorded to her male peers.

Notably, Charlotte Pommer was never a member of the *Anatomische Gesellschaft* (German Anatomical Society) or the *Deutsche Gesellschaft für Pathologie* (German Society of Pathology). This research demonstrates that membership in such societies is not necessarily a proxy for the historical significance of the individuals concerned. Subsequently, this study forms part of a broader investigation into the *Anatomische Gesellschaft* and its female members, a line of inquiry that future publications and projects should pursue.

Her later writings and correspondence, particularly the letters to Golo Mann between 1978 and 1989, add a profound self‐reflective dimension to the archival record. In these letters, Pommer articulated a concept she termed the war of “Germans against Germans,” a phrase that also appears in her manuscript,^3^ ^(p118)^ and through which she contextualized her wartime and post‐war experiences as an enduring moral and ideological conflict within German society itself. Her comments on abortion, expressed in religious‐moral language consistent with her conversion to Catholicism, link this “inner war” to broader questions of humanity amidst the medical and social transformations of the 1970s and 80s.

Her exchanges with Mann further reveal an ongoing struggle to define individual guilt and collective responsibility: she explicitly rejected Mann's categorization of resistance fighters and asserted instead a deeply personal moral accountability shaped by her medical and wartime experiences. These reflections, culminating in her quotation of Walther von der Vogelweide's “peace and justice are sorely wounded” in her final letter, suggest a sustained preoccupation with the ethical disintegration of her time and its lasting effects on her self‐conception as both physician and witness. Her vehement anti‐communism—a force by which she felt persecuted—mirrors the intensity of her resistance against the Nazi regime, a trait she shared, presumably by chance, with Albrecht Tietze,^12^ ^(p26)^ highlighting a worldview shaped by their shared wartime experiences.

This enduring conflict may also explain her restless post‐war biography. Her frequent relocations across continents—from Ethiopia and India to North America and various European cities—suggest a search for a place where she could find peace, both professionally and morally. Unable or unwilling to settle in a society she viewed as morally compromised, she remained suspended between guilt and faith, defiance and resignation, individual conscience and national belonging. Her late self‐representation thus complements and deepens the interpretation of her earlier professional trajectory: a life marked by conscientious autonomy, religious introspection, and an enduring conflict between moral conviction and institutional conformity—first under National Socialism, and subsequently in a post‐war Germany whose treatment of abortion rights and unresolved “Nazi legacy” she could neither accept nor escape.

## CONCLUSION: A SELF‐DETERMINED LIFE

Charlotte Pommer (1914–2004) emerges from the archival record as an early—and possibly the earliest clearly documented—example of female de facto leadership at a German anatomical institute. Her case stands alongside that of Clara Zawitsch‐Ossenitz in Graz^37^ but appears to be the first such instance within Germany itself. A definitive answer awaits the comparative national survey this study has identified as a research desideratum.

Beyond this institutional dimension, the new findings presented here—on her family, her international career, her correspondence with Golo Mann, and her late work on Maupertuis—reveal a biography that cannot be reduced to a single moment of resistance or a single professional role. What makes Pommer significant is precisely the conjunction of a documented pioneer of women in German anatomy with a self‐authored testimony of moral struggle. Her life thereby bridges the institutional history of post‐war German medicine and the long afterlife of National Socialism in individual biographies—a perspective that, despite the absence of a surviving photograph, deserves a permanent place in the historiography of women in science and of conscientious dissent under dictatorship.

## AUTHOR CONTRIBUTIONS

**Tim S. Goldmann:** Investigation; writing – original draft; methodology; data curation.

## Data Availability

Data sharing not applicable to this article as no datasets were generated or analysed during the current study.

## Footnotes

^a^
According to his two children, with whom the author was in contact.
